# Integrated Quality by Design Approach for Developing Nanolipidic Drug Delivery Systems of Olmesartan Medoxomil with Enhanced Antihypertensive Action

**DOI:** 10.34172/apb.2020.046

**Published:** 2020-05-11

**Authors:** Jagdish Kumar Arun, Rajeshwar Vodeti, Birendra Shrivastava, Vasudha Bakshi

**Affiliations:** ^1^School of Pharmaceutical Sciences, Jaipur National University, Jagatpura, Jaipur, Rajasthan-302 017, India.; ^2^School of Pharmacy, ANURAG Group of Institutions, Venkatapur (V), Ghatkesar (M) Medchal (Dist.), Hyderabad, Telangana-500 038, India.

**Keywords:** Bioavailability, Nanoemulsion, Optimization, Experimental design, Pharmacokinetics

## Abstract

***Purpose:*** The present work endeavors to report a systematic approach of developing the lipidic self-nanoemulsifying formulation of olmesartan medoxomil (OMT) on the principles of Quality by Design (QbD).

***Methods:*** For preparing the self-nanoemulsifying formulation, a mixture of oil, surfactant and cosurfactant were used as vehicles. The excipients were selected after screening by solubility as well as pseudoternary phase titration studies. Mixture design was adopted for systematic optimization of the composition of nanolipidic formulations, which were evaluated for smaller globule size, stable zeta potential and lower values of polydispersity index. The optimized liquid self-nanoemulsifying formulation was identified using numerical and graphical optimization techniques, followed by validation of the experimental model. Solidification of self-nanoemulsifying formulation was carried out using porous carriers, and then optimized on the basis of oil adsorption potential, powder flow property and drug release performance. Pharmacokinetic study was performed in male Wistar rats for evaluating the drug absorption parameters. All the experimental data obtained were subjected to statistical analysis using oneway ANOVA followed by post hoc analysis using Student’s t test.

***Results:*** The optimized liquid self-nanoemulsifying formulation showed globule size <100 nm, emulsification efficiency <5 minutes and*in vitro* drug release >85% within in 30 minutes. Further, the solid SNEDDS formulation was effectively formulated using Neusilin US2 with maximum oil adsorption capacity and good micromeritic properties. Pharmacokinetic evaluation indicated 4 to 5-folds increase (*P* <0.05) in the values of C_max_, AUC, and reduction in T_max_ from the nanoformulations vis-à-vis the marketed formulation.

***Conclusion:*** Overall, the developed nanolipidic formulation of olmesartan indicated superior efficacy in augmenting the drug dissolution and absorption performance.

## Introduction

Quality by Design (QbD), a concept proposed by American quality expert, J.M. Juran for systematic planning and execution of manufacturing operations for avoiding quality crisis.^1,2+^ Later, United States Food and Drug Administration (USFDA) adopted the Juran’s concept of QbD into pharmaceutical drug product development. QbD approach provides a platform for systematic development of the drug products with predefined objectives for rational selection of the product and process parameters influencing the end-product quality. Hence, the product development as per QbD paradigm provides enhanced product and process understanding, and robust performance.^3-5^

Olmesartan medoxomil (OMT), a potential antihypertensive agent, inhibits the synthesis of angiotensin II by blocking the angiotensin type I receptor. Despite its efficacy, it shows poor aqueous solubility and high lipophilicity, as the major rate-limiting factors for low and inconsistent oral bioavailability.^6,7^ Several literature reports have demonstrated the application different solubility enhancement technologies for improving biopharmaceutical performance of the drug.^8-12^ However, beyond solubility, many other rate-limiting factors tend to affect the oral bioavailability of OMT. Some of them are first-pass effect and terminal metabolism by gut enzymes. Thus, a novel formulation strategy used must address the aforementioned challenges for getting maximal therapeutic benefits of the drug.

Lately, self-nanoemulsifying drug delivery systems (SNEDDS) have attracted much interest of researchers as one of the extensively investigated formulation approaches for number of BCS class II and IV drugs.^13^ Constituted of a blend of oils, emulgents and cosolvents, SNEDDSs have unparalleled potential not only for augmenting the solubility of the drugs but also by overcoming the biological metabolism of drugs. Overall, this ultimately increases the oral bioavailability of the drugs to several folds.^14,15^ Besides, SNEDDS provide added advantage for ease of preparation, scale-up and prolonged stability for months.

The present research work, therefore, was undertaken as per the QbD framework, in order to carry out the experimental design-guided development, optimization and evaluation of the nanoformulation of OMT. Besides, the optimized liquid nanoformulation formulation was converted into solid, which was further optimized on the basis of desirable formulation attributes.

## Materials and Methods

OMT was supplied from M/s Cipla Pharmaceuticals (Baddi, India). Various lipids used for formulation development were obtained from M/s Gattefosse (Cedex, France) and M/s Abitec Inc. (Janesville, USA). The emulgents and cosolvents used were also purchased from M/s Fischer Scientific and M/s SD Fine Chemicals (Hayana, India). The porous carriers used in the study were provided by M/s Evonik GmbH (Dresden, Germany) and M/s Gangwal Chemicals (Mumbai, India).

### Target product profile (TPP)

TPP includes the entire drug product development plan enlisting the key quality attributes of the drug product, which have direct link with patient safety and efficacy.^16,17^ TPP was defined for target liquid SNEDDS formulation of OMT, in order to augment the oral bioavailability of the drug. The elements of typical TPP include type of formulation, strength, delivery route, drug release and pharmacokinetics, stability and packaging attributes.

### Critical quality attributes (CQAs)

Out of the TPPs, some important quality attributes were selected as the CQAs on the basis of their criticality of influence on patients benefit. For the target SNEDDS formulation, we earmarked *viz*. particle size (PS, indicator of formulation stability and drug absorption potential through GI tract), time for emulsification (ET, crucial for rapid drug dissolution in the gastrointestinal fluid), mean time for dissolution (TD) and dissolution efficiency (DE) (pivotal for drug release from the formulation).

### Formulation quality risk management

Fish-bone diagram was drawn by highlighting a list of causal factors such as drug substance, excipients, machines, measurement techniques and environment milieu, and their sub-causes on the CQAs of SNEDDS. Further, a risk matrix was created by giving green, yellow and red color coding to portray low, medium and high level of risk, respectively, for each of the formulation and/or process attributes.^18,19^

### Screening of formulation excipients

Solubility of the drug was estimated in various lipids like Peceol, Maisine 35-1, Capmul MCM, ethyl oleate, Lauroglycol 90 and Lauroglycol FCC. Also, solubility evaluation was carried out in various surfactants such as Tween 20, Tween 80, Acrysol K-140, Acrysol EL-135, and cosolvent, Transcutol HP. Excess quantity of drug was added to each of the excipients and kept on a mechanical shaker (Rivotek, Riviera Glass Pvt. Ltd., Mumbai, India) with water bath maintained at 37 ± 0.5°C for 72 hours. Vials were examined time to time for solubilization of the drug and subsequently drug was added if required. All the excipients were transferred into centrifuge tubes (Spinwin MC02, Tarsons Pvt Ltd., Kolkata, India) for separation of the undissolved drug, while actual amount of drug solubilized was analyzed spectrophotometrically (UV-Vis Spectrophotometer, Labindia Ltd., Mumbai, India) at 243 nm (i.e., absorption maxima of the drug) from the supernatant fraction.^20^

Aqueous titration procedure was used for constructing the pseudoternary phase diagrams. Lipid with maximum drug solubilization capacity was titrated with surfactant/cosolvent mixtures (S_mix_) in the ratio between 1:9 and 9:1. Various S_mix_ ratios explored were 1:0, 1:1, 2:1 and 3:1, thus selecting the ratio with maximal region for nanoemulsion formation.^21^

### Preparation of liquid SNEDDS formulation

The preparation of liquid SNEDDS was attempted using simple admixture method, where selected oil, surfactant and cosolvent were added one by one to the clean and dry culture tubes. The contents were thoroughly mixed under mild heating at 40 ± 2^o^C with the help of vortex mixer (Cyclomixer CM 101, Remi Elektrotechnik Ltd., Mumbai, India) in order to achieve a homogeneous mixture of the excipients. Then, accurately weighed amount of the drug was added to the excipient mixture and thoroughly mixed to solubilize it.^22^ The formulations were stored in cool and dry place till further analysis.

### Systematic formulation optimization studies

Experimental designs are the important elements of QbD, which helps in optimizing the formulation composition and process parameters. Among the various design available, mixture response surface designs are very useful in optimizing the nanoemulsion where sum total of all the components are equal to 100%.^23,24^ Hence, 3-factor and 3-level containing D-optimal response surface design with mixture components was adopted for optimizing the SNEDDS composition. Design Expert^®^ 9.0.1 (M/s Stat-Ease Inc., Minneapolis, USA) was used for generating the experimental trials, where quantities of Capmul MCM, Tween 80 and Transcutol HP were taken as the factors. A total of 16 trial formulations were prepared including five replicates of the center point trial, thus evaluated for the formulation CQAs.

### Characterization of the liquid SNEDDS formulations Globule size measurement

The SNEDDS formulations were diluted with distilled water and globule size was measured by dynamic light scattering technique using Zetasizer ZS-90 (Malvern Instruments, Worcestershire, UK). Mean globule size of nanoemulsion formed after dilution as Z-average diameter was noted.

### Emulsification time determination

The SNEDDS formulations were diluted with distilled water and time required for nanoemulsion formation was noted.

### Transmission electron microscopy (TEM)

TEM imaging of the optimized liquid SNEDDS formulation was carried out after 100-fold dilution, followed by negative staining with 1% phosphotungstic acid solution on a gold plate. Then, images were captured under electron microscope (JEM-2100 F, Jeol, Tokyo, Japan) for morphological characterization of the emulsion globules.

### Drug release evaluation

It was conducted using dialysis bag (MW 1 kDa) diffusion method, where the SNEDDS formulations were exposed to simulated gastric and simulated intestinal fluids (250 mL), both containing 0.5% sodium lauryl sulfate for the period of 1 h and 2 h, respectively.^22,25,26^ SNEDDS loaded with 20 mg drug were diluted in 1 ml of SGF and filled in the dialysis bags. The blank formulations were also run for each of the studied the formulations to nullify the interference of excipients with drug during analysis. Similarly, the drug release from pure drug suspension was also performed for comparative evaluation with the SNEDDS formulations.^27^ The samples were collected at periodic time intervals and an equal volume of fresh medium was added. The collected samples were analyzed using HPLC (Alliance 2695, Waters, Massachusetts, USA) method of the drug and cumulative amount of drug release versus time was calculated. The chromatographic analysis of the drug was performed using the reported procedure on a C_18_ column using acetonitrile and water (containing 0.1% orthophosphoric acid, pH 3.5) in 40:60 (v/v) as mobile phase at a flow rate of 1.0 mL/min with UV detection at 243 nm.^28^ The obtained drug release data was then subjected to modeling to fit with various kinetic models as reported in literature and best suited model governing the mechanism of drug release from the SNEDDS formulation was identified.^29,30^

### Modelization, data analysis and optimum search

The modelization of experimental data was conducted by linear regression analysis. Best fit model was selected by virtue of its statistical validity for the model terms and evaluating parameters like good correlation coefficient, lower residual sum of square and lower lack of fit.^31,32^ Moreover, the developed mathematical equation was observed for the interaction terms and their net resultant influence on the studied CQAs. Various model plots as diagnostic tools of the design were analyzed to check the feasibility and suitability of the data. In addition, 3D- and 2D-plots were studied for analyzing the factor-response relationships. The optimum formulation was identified by numerical and graphical search techniques. Validation was performed by comparing the closeness between the predicted values with experimental values of the CQAs.^33^

### Evaluation of the optimized liquid SNEDDS formulation

The optimized SNEDDS formulation was subjected to size measurement and comparative *in vitro* drug release analysis with the marketed immediate release tablet formulation (Olmecip, M/s Cipla Ltd., Mumbai, India) and pure drug suspension.

### Development and evaluation of the solid SNEDDS formulation

As widely reported in the literature, adsorption technique was employed for preparing the solid SNEDDS. The optimized liquid SNEDDS formulation was subjected wet mixing with Aerosil 200 in a mortar-pestle, and passed through 30 mesh sieve to obtain the uniform powder mass. The weight quantity of carrier material required for adsorption of the oily formulation was noted and powder was subjected to the micromeritic characterization.

### Pharmacokinetic evaluation studies

The pharmacokinetic evaluation of the SNEDDS was conducted in unisex Wistar rats under fasting condition. The rats were randomly distributed into four groups, with six animals in each group. Group I and II rats were given with solid and liquid SNEEDS formulations, while group III and IV rats were administered with marketed formulation and pure drug suspension, respectively. All the formulations were administered with the animal dose of the drug (20 mg) equivalent to the body weight of rats. After dosing, blood samples (~0.2 mL) were withdrawn by puncturing the tail vein and centrifuged to collect plasma for HPLC analysis. The chromatographic analysis of the drug was performed using the reported procedure on a C_18_ column using acetonitrile and water (containing 0.1% orthophosphoric acid, pH 3.5) in 40:60 (v/v) as mobile phase at a flow rate of 1.0 mL/min with UV detection at 243 nm.^28^ The developed method was translated to bioanalytical level for estimation of the drug in rat plasma as per the reported procedure.^32,34,35^ All the blood samples were analysed using the HPLC method and plasma concentration of the drug at various time points were measured. Various pharmacokinetic parameters were calculated using non-compartmental analysis method for different groups of treatments.

### Statistical data analysis 

All experimental data obtained were subjected to statistical analysis using ANOVA, followed by post-hoc analysis using Student’s *t* test at 5% level of significance.

### Accelerated stability study

The optimized liquid and solid SNEDDS formulations were subjected to accelerated stability study for six months using a stability chamber (TH 200G, Thermolab, Thane, India). Both the formulations were filled in capsules and packed in a glass bottle with cotton plug and kept in the stability chamber maintained at 40°C temperature and 75% relative humidity. The samples were removed from stability at 0, 1, 2, 3 and 6 months time periods, and evaluated for emulsification efficiency, globule size, zeta potential and drug release in 15 minutes.

## Results and Discussion

### Target product profile (TPP)

Supplementary data Table S1 gives account of TPP elements of the liquid SNEDDS of OMT, which includes formulation type, clinical dose, drug release nature, intended route of delivery, pharmacokinetics, packaging and stability requirements.

### Critical quality attributes (CQAs)

Out of TPP elements, the key formulation attributes were identified as the CQAs for liquid SNEDDS. Supplementary data Table S2 gives account on rational justification for selection of CQAs and their influence on the biopharmaceutical performance of the liquid SNEDDS.

### Excipients screening

**Figure 1:**
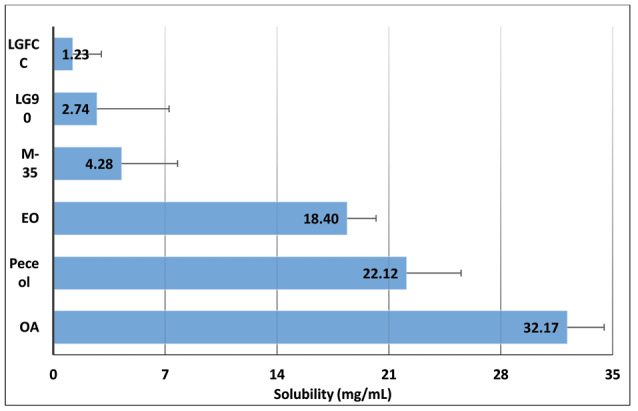
Equilibrium solubility data of OMT in lipids. Data expressed as mean ± SD (n=3). OMT: Olmesartan medoxomil; OA: Oleic acid, EO: Ethyl oleate, M-35: Maisine 35-1, LG90: Lauroglycol 90, LGFCC: Lauroglycol FCC.

**Figure 2:**
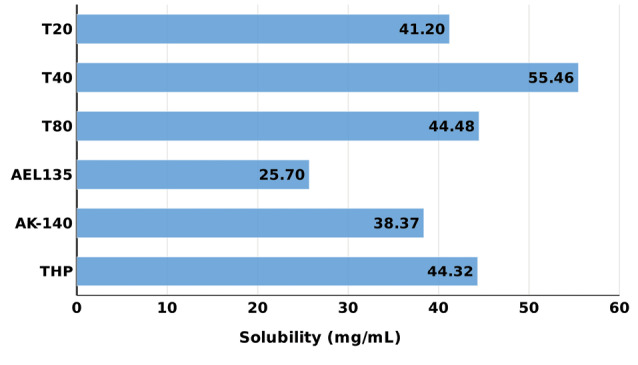
Equilibrium solubility data of OMT in surfactants and cosolvents. Data expressed as mean ± SD (n=3). OMT: Olmesartan medoxomil; THP: Transcutol HP; AK-140: Acrysol K-140, AEL135: Acrysol 135, T80: Tween 80; T40: Tween 40, T20: Tween 20.

Figure 1 shows the bar chart depicting the drug solubility in various lipids, which were found to be in the order: Lauroglycol FCC < Lauroglycol 90 < Maisine 35-1 < ethyl oleate < Peceol < Capmul MCM. Capmul MCM was chosen as the lipid with maximum solubility for the drug. Similarly, Figure 2shows the bar chart depicting the solubility of drug in surfactants and cosolvents, which were found to be in the order: Acrysol EL-135 < Acrysol K-140 < Tween 20 < Tween 80 < Transcutol HP.

### Pseudoternary phase diagrams

**Figure 3:**
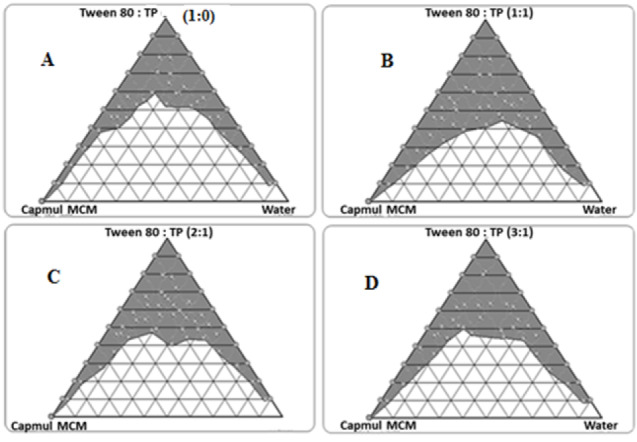
Pseudoternary phase diagrams depicting nanoemulsion region with Smix ratios (A) 1:0, (B) 1:1, (C) 2:1 and (D) 3:1

Figure 3 depicts the images showing pseudoternary phase diagrams for Capmul MCM titrated with Tween 80-Transcutol HP (S_mix_) at 1:0, 1:1, 2:1 and 3:1 ratios. In all S_mix_ combinations, we observed good nanoemulsion region indicating good emulsification characteristics of the selected surfactant and cosolvent. Increasing the amount of surfactant in S_mix_ also showed gradual increase in the area of nanoemulsion region. Among the studies S_mix_ combinations, 1:1 ratio was chosen for the formulation of SNEDDS.

### Characterization of the liquid SNEDDS

Supplementary data Table S3 summarizes the values of CQAs, as the physical and dissolution parameters of the prepared liquid SNEDDS prepared as per the mixture experimental design.

### Globule size measurement 

All the formulations showed globule size in the range between 45 and 99 nm. The smaller size less than 100 nm thus confirmed the nano lipidic nature of the prepared formulation, which are inconsonance with the literature reports.^36^

### Emulsification time determination

All the formulations showed emulsification time in the range between 103 and 184 seconds. This confirmed good self-emulsification efficiency of the prepared nano lipidic formulation, ostensibly ascribed to the characteristic nature of the excipients.^37^

### Transmission electron microscopy (TEM)

The inset of Figure 3AillustratesTEM images of the liquid SNEDDS formulations with dark spots as the nanoemulsion globules. The result observed is inconsonance with the literature reports.^36,37^

### Drug release performance

**Figure 4:**
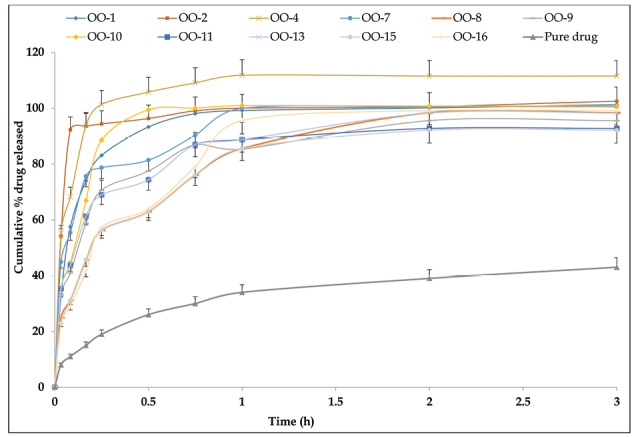
Cumulative drug release profile of OMT from different liquid SNEDDS in dialysis membrane with molecular cut-off weight of 1 kDa. Data expressed as mean±SD (n=3). OMT: Olmesartan medoxomil; SNEDDS: Self-nanoemulsifying drug delivery systems..

Figure 4 portrays the release profiles of OMT from liquid SNEDDS formulations. More than 90% drug was released from all the SNEDDS formulations within 1 h without any lag-phase. While, drug release profile from pure drug suspension showed maximum of 42% drug release in 1 hour. Further, the dissolution was continued for 3 hours, in case of pure drug suspension also indicated incomplete drug release. The net release of drug was up by 2.5-fold by SNEDDS formulation as compared to the suspension of pure drug. Besides, the calculation of mean time of dissolution indicated values ranging between 3.2 and 18.6 minutes, while efficiency of dissolution indicated values between 20% and 34%, respectively for the SNEDDS. These results further confirmed faster drug release characteristics of the nanolipidic formulation of OMT.^20,38^

### Modeling and optimization data analysis

The coefficients of each model term generated as per the Eq. (1) for CQAs has been listed in Supplementary data Table S4.

Y = β_1_X_1_ + β_2_X_2_ + β_3_X_3_ + β_4_X_1_X_2_ + β_5_X_2_X_3_ + β_8_X_1_X_2_(X_1_-X_2_) + β_n_X_n_X_m_(X_n_-X_m_) Eq. (1)

where, Y indicates the CQA; β_1_ to β_5_ are the coefficient values of various model terms; X_1_, X_2_, X_3_ indicates the CMAs used for optimization studies

The magnitude of polynomial coefficients of the model terms in the polynomial equations of the CQAs confirmed factor interaction effects. High values of correlation coefficients ranging from 0.9414 and 0.9998, and insignificant values of lack of fit indicated goodness in the model fitting.

### Response surface analysis

**Figure 5:**
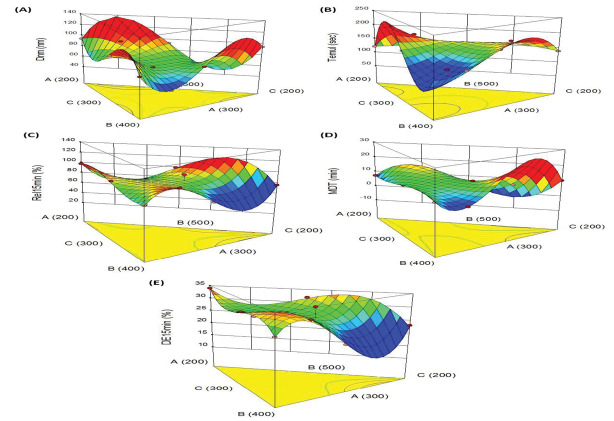
3D-Response surface plots indicating the relationship between the factors A, B and C on the response variables, (A) particle size, (B) time required for emulsification, (C) drug release in 15 min, (D) mean dissolution time, (E) dissolution efficiency in 15 min. Dnm: Globule size, Temul: Emulsification efficiency, Rel15min: Cumulative %drug release in 15 minutes, MDT: Mean dissolution time, DE15min: Dissolution efficiency in 15 minutes.

The understanding of the dependence, inter-dependence and co-variation among the variables studied and their interactions is facilitated by constructing response surface diagrams to map the responses over the entire experimental domain.^39^ The 3D- and 2D-plot for various CQAs are shown in Figure 5.

Figure 5A illustrates the 3D graph indicating influence of Capmul MCM, Tween 80 and Transcutol HP on particle size. Among the CMAs, Tween 80 shows curvilinear trend on size, where high level of Tween 80 helps in reducing the size. However, increase in Capmul MCM and Transcutol HP levels yielded linear escalation in the size from minimum to maximum levels. The response surface graph shown in Figure 5B is for time of emulsification. This portrays a “saddle shaped” structure, revealing significant influence of Capmul MCM and Transcutol HP on time, whereas Tween 80 shows quite negligible effect.

Figure 5C depicts the inverted “saddle shaped” 3D graph for drug release behaviour. This shows a sharp dip in drug release profile upon increase in the concentrations of Capmul MCM and Tween 80 while the effect of Transcutol HP on Rel_15min_ shows quite negligible influence.

The typical response surface plots for MDT depicted in Figure 5D shows the prominent influence of Capmul MCM and Transcutol HP. The curvilinear trend can be observed for MDT upon increasing the levels of both of these constituents. However, the effect of Tween 80 on MDT was found to be quite negligible.

Like drug release, analogous 3D plot was revealed for the influence efficiency of dissolution. Figure 5E depicts the maximal values of DE_15min_ at lower levels of Capmul, high levels of Tween and Transcutol. As the prepared formulations exhibited quite higher values of Rel_15min_, the values of DE_15min_ also show an increasing trend in close proximity with the faster drug release characteristics.

### Selection of the optimum SNEDDS formulation

**Figure 6:**
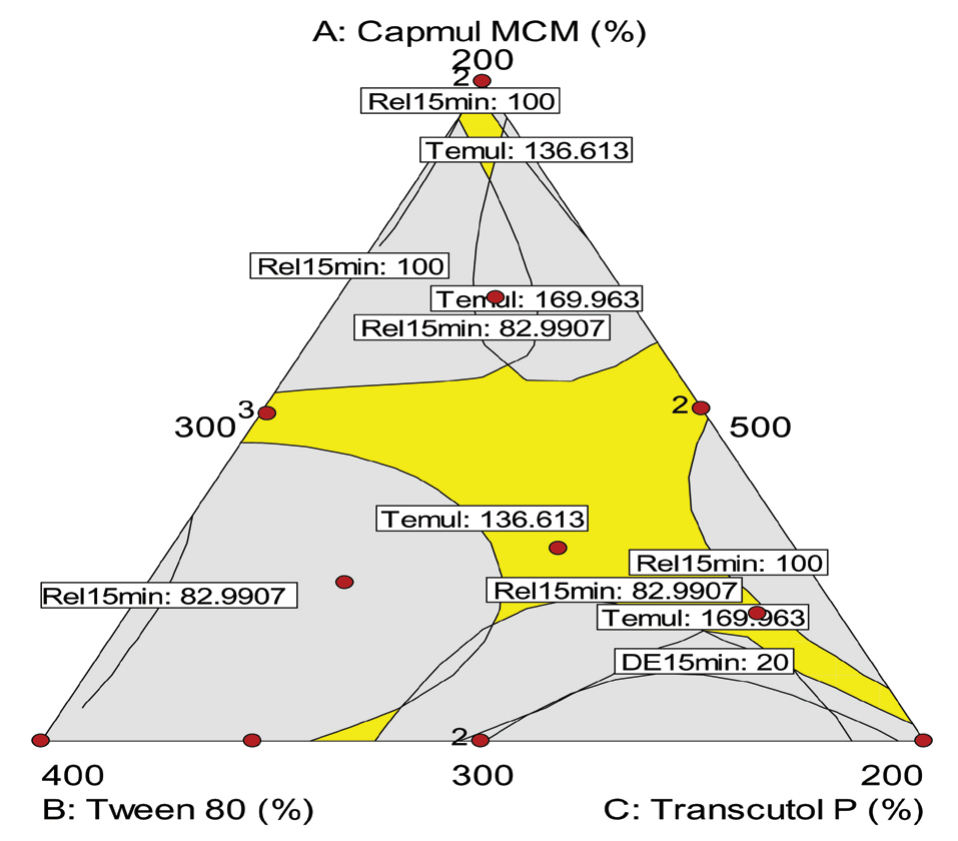
Design space overlay plot for optimized liquid SNEDDS of OMT. OMT: Olmesartan medoxomil; SNEDDS: Self-nanoemulsifying drug delivery systems, Dnm: Globule size, Temul: Emulsification efficiency, Rel15min: Cumulative %drug release in 15 minutes, MDT: Mean dissolution time, DE15min: Dissolution efficiency in 15 minutes.

The optimum formulation was identified out on the basis of various goals, i.e., smaller the particle size and lesser the time required for emulsification, and faster the drug release profile, higher the mean dissolution time and efficiency for dissolution. The desirable region was also selected using overlay plotting, as shown in Figure 6. The optimized formulation contained, i.e., Capmul MCM (393 mg): Tween 80 (423 mg): Transcutol HP (184 mg), as indicated by flagged point in the figure, along with predicted values of various CQAs.

### Evaluation of the optimized formulation

#### Globule size and zeta potential

**Figure 7:**
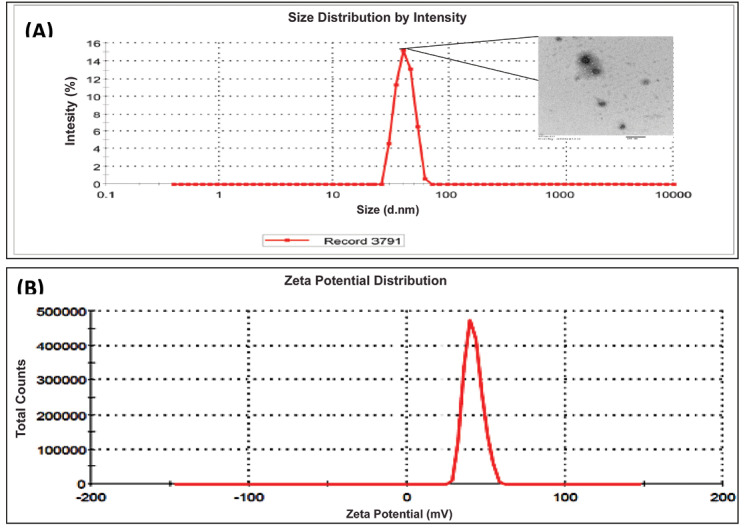
(A) Globule size and TEM image (B) Zeta potential distribution of optimized liquid SNEDDS [SNEDDS: Self-nanoemulsifying drug delivery systems]

Figure 7A depicts globule size distribution of the optimized liquid SNEDDS with mean globule 64.2. The obtained size in the nanometric range fulfilled the target particle size of the developed formulation.

### Zeta potential

The magnitudes of zeta potential of the optimized liquid SNEDDS was found to be -25.4 mV (Figure 7B). The obtained negative magnitude of zeta potential fulfilled the requirement for the developed formulation.

### Evaluation of solid SNEDDS

#### Micromeritic characterisation

Supplementary data Table S5enlists the micromeritic properties of solid SNEDDS, where true density ranged from 1.29 to 1.67 g.cm^-3^, bulk density ranged from 0.20 and 0.27 g.cm^-3^, and tapped density ranged from 0.21 and 0.49 g.cm^-3^, respectively. Based on these micromeritic properties, the best porous carrier was selected.^40,41^

### Flow and compaction properties

The flow and compaction parameters of solid SNEDDS are enlisted in Supplementary data Table S6. Solid SNEDDS formulations showed good flow behavior, as indicated by angle of repose ranged from 22.6 to 39.3 degree, and Carr’s index ranged 17.2 from 39.5. All these parameters construed good overall flow behaviour of the carriers used for preparing solid SNEDDS, which can provide desired compaction and compression ability.^40,42^

### Drug release evaluation

The rate and extent of drug release from the solid SNEDDS formulations was found to be analogous with the liquid SNEDDS (*P* < 0.05). Moreover, both the formulations exhibited immediate drug release nature (>85%) within 30 minutes and complete drug release in 60 minutes, thus fulfilled the expected drug release performance. The obtained data for the release analysis of liquid and solid SNEDDS are inconsonance with the literature reports published by other authors.^38,42^ No statistically significant difference (*P* > 0.05), however, was observed in the drug release profile of liquid and solid SEDDS. Additionally, release characterization of the pure drug showed incomplete drug release only upto 43% into the dissolution medium, which can be ascribed to the poor aqueous solubility of the drug as reported in literature.^22,43^

### Drug release kinetics modeling

The release kinetic analysis of the obtained drug release data indicated best model fitting as per first-order model by Fickian diffusion mechanism, which can be attributed to the fluidic nature of the prepared formulation. The observed findings are inconsonance with the literature reports.^38,44^

### In vivo pharmacokinetic studies

**Figure 8:**
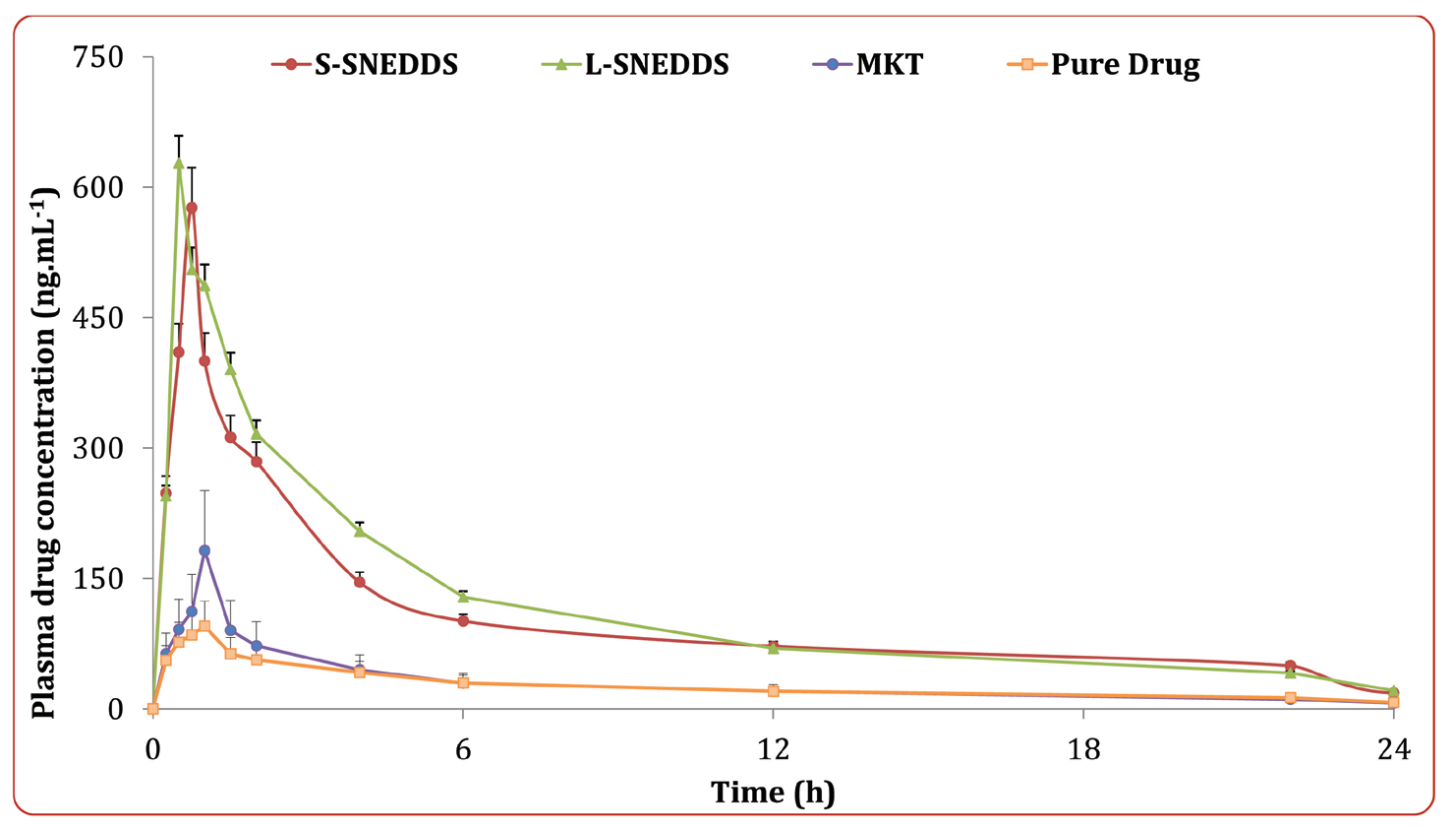
Plasma concentration versus time profile of OMT from the optimized solid SNEDDS, liquid SNEDDS, MKT formulation and pure drug suspension; while the inset depicts percent alteration in various parameters from the treatment formulations vis-à-vis the pure drug. Data is represented as mean ± 1SD (n=6). OMT: Olmesartan medoxomil; L-SNEDDS: Liquid Self-nanoemulsifying drug delivery systems; S-SNEDDS: Solid Self-nanoemulsifying drug delivery systems; MKT: Market formulation.

The mean plasma concentration with respect to time profile of the drug obtained after oral administration of the different treatment formulations is shown in Figure 8. The plasma concentration data of liquid SNEDDS showed nearly 5.58 and 4.21-folds extension in C_max_ and AUC values, and 0.54-folds drop in T_max_ values, while solid SNEDDS revealed nearly 5.06 and 3.87-folds up in C_max_ and AUC values, and 0.31-fold drop in T_max_ values, respectively. These results ratify distinct progress in the oral bioavailability of drug from SNEDDS formulations vis-à-vis pure drug (*P* < 0.0001), plausibly owing to the superior drug absorption characteristics as a function of avoidance of hepatic first-pass effect, improvement in the aqueous drug solubility, dissolution rate and intestinal drug permeability.^45-48^ Moreover, the pharmacokinetic parameters for the solid SNEDDS revealed quite analogous results with those of the liquid SNEDDS (*P* > 0.05). This demonstrated that adsorption of the liquid SNEDDS onto the solidifying carriers showed no significant change in the bioavailability parameters, except a mild extension in the values of T_max_ and AUC for solid SNEDDS as compared to the liquid SNEDDS. The observed findings were quite inconsonance with the formulation characteristics reported in literature.^43^

### Accelerated stability study

Supplementary data Table S7 provides the values of various physical and dissolution parameters of the liquid and solid SNEDDS formulations. Statistically insignificant (*P* > 0.05) difference was observed in the parameters, i.e., emulsification time, globule size, zeta potential and drug release during the six-month evaluation period. This construed highly stable and robust nature of the prepared formulations even under stress conditions of high temperature and humidity.

## Conclusion

The present research work ratifies the successful development of SNEDDS formulations of OMT. Use of QbD approach was very useful in optimizing the formulating composition and identifying the best formulation with CQAs matching the desired limits. As envisioned, the SNEDDS exhibited good *in vitro* and *in vivo* performance. Thus, it can be concluded that SNEDDS developed using porous carriers has industrial importance with respect to its applicability for other drugs having oral bioavailability challenges.

## Ethical Issues

All the animal studies performed in the preset work were carried out prior approval of the study protocol (Reference No. I/IAEC/AGI/025/2018) from the Institutional Ethics Committee, Anurag Group of Institutions, Hyderabad, India. All the studies were carried out as per the guidelines of Committee for the Purpose of Control and Supervision of Experiments on Animals (CPCSEA), Government of India.

## Conflict of Interest

Authors declare no conflict of interest.

## Supplementary Materials


Supplementary file 1 contains Tables S1-S7.
