# Supplementary material for: Integrated Quality by Design Approach for Developing Nanolipidic Drug Delivery Systems of Olmesartan Medoxomil with Enhanced Antihypertensive Action
Source: Adv Pharm Bull. 2020 May 11;10(3):379–88. doi: 10.34172/apb.2020.046 (PMC7335990; doi:10.34172/apb.2020.046)
Supplement: Supplementary file 1 — contains Tables S1-S7. [file apb-10-379-s001.pdf]

Supplementary Data

Table S1. QTPP elements for liquid SNEDDS of OMT

QTPP Elements	Target	Justification
Dosage form	SNEDDS	Selection of lipid-based self-nanoemulsifying system helps in the oral bioavailability enhancement of poorly bioavailable drug, OMT
Dosage type	Immediate release	Faster onset of action leads to enhanced therapeutic benefits
Dosage strength	20 mg	Unit dose of OMT incorporated in a single formulation of L-SNEDDS
Route of administration	Oral	Recommended route for delivery of OMT for the management of hypertension
Pharmacokinetics	Higher C_{max} and AUC	Required for achieving higher drug levels into the systemic circulation for enhanced therapeutic action
Packaging	Hard gelatin capsules	L-SNEDDS can be easily delivered in hard gelatin capsules for improved patient compliance, portability and manufacturing ease
Stability	At least 24 months	To maintain therapeutic potential of the drug during storage period

OMT: Olmesartan medoxomil, SNEDDS: Self-naoemulsifying drug delivery systems

Table S2. CQAs of liquid SNEDDS of OMT and rational justifications for them

Quality attributes	Target	Is this a CQA?	Justification(s)
Globule size (D_{nm})	< 100 nm	Yes	Smaller globule size allows easier penetration through GI epithelial lining and paracellular pathways; hence was regarded as highly critical.
Emulsification time (T_{emul})	< 5 min	Yes	Lower values of emulsification time facilitate the formation of nanoemulsion; hence was taken up as highly critical.
%Drug release in 15 min (Rel_{15min})	100%	Yes	It indicates performance of the dosage form during early dissolution phase; therefore, was considered as critical.
Mean dissolution time (MDT)	Low	Yes	It is an indicator of faster and complete release of the drug in the dissolution medium; thus was taken up as highly critical.
%Dissolution efficiency in 15 min (DE_{15min})	100%	Yes	It provides information on *in vitro* dissolution performance of the dosage form during dissolution phase; therefore was considered as critical.

CQA: Critical quality attributes, OMT: Olmesartan medoxomil, SNEDDS: Self-naoemulsifying drug delivery systems

Table S3. Values of CQAs of liquid SNEDDS of OMT prepared as per the D-optimal design

Formulation Code	Critical Material Attributes (CMAs)			Critical Quality Attributes (CQAs)				
	Capmul MCM (mg)	Tween 80 (mg)	Transcutol HP (mg)	Globule size (D_{nm})	T_{emul} (sec)	Rel_{15min} (%)	MDT (min)	DE_{15min} (%)
F1	276	446	278	72.22	103.23	84.08	8.61	28.23
F2	300	424	276	45.34	124.01	100.04	4.43	31.34
F3	200	500	300	94.97	122.07	98.56	7.34	34.67
F4	250	500	250	93.88	151.98	99.12	6.25	32.09
F5	200	500	300	94.05	122.06	98.87	7.37	34.88
F6	250	450	299	86.11	155.11	92.56	10.29	30.94
F7	250	450	299	86.01	155.08	92.88	10.22	30.11
F8	300	450	250	76.82	175.65	64.34	17.32	20.21
F9	300	400	300	78.77	113.79	80.06	10.35	27.33
F10	271	473	256	67.91	141.23	99.91	11.41	31.43
F11	233	485	282	98.05	184.47	75.16	13.20	25.58
F12	250	500	250	93.05	151.68	99.73	6.29	32.78
F13	300	500	200	94.82	137.91	75.68	10.11	23.65
F14	250	450	299	86.93	155.95	92.93	10.24	30.83
F15	281	491	228	65.55	172.55	89.68	7.26	24.92
F16	300	450	250	76.71	175.45	64.55	17.31	20.99

CQA: Critical quality attributes, OMT: Olmesartan medoxomil, SNEDDS: Self-naoemulsifying drug delivery systems

Table S4. Polynomial and correlation coefficients obtained as per the quadratic model for CQAs of the liquid SNEDDS of OLM

Coefficient codes	Polynomial coefficients for response variables				
	D_{nm}	T_{emul}	Rel_{15min}	MDT	DE_{15min}
β_1	94.71	123.50	76.74	12.32	22.61
β_2	83.60	131.50	95.80	7.38	30.14
β_3	89.54	137.00	98.68	4.51	31.08
β_4	-42.68	24.00	-23.07	18.26	0.49
β_5	-38.13	-71.00	15.55	-3.44	17.73
β_6	-97.40	151.00	-30.57	-0.050	-25.32
β_7	-2254.23	-2677.20	2768.82	86.72	947.70
β_8	406.56	-1186.80	585.26	-40.13	174.47
β_9	1674.46	-1018.20	773.67	-92.77	234.69
R	*0.9426*	*0.9414*	*0.9998*	*0.9701*	*0.9655*
p-Value	*<0.01*	*<0.01*	*<0.001*	*<0.001*	*<0.001*

CQA: Critical quality attributes, OMT: Olmesartan medoxomil, L-SNEDDS: Liquid self-naoemulsifying drug delivery systems, D_{nm}: Globule size, T_{emul}: Emulsifcation time, Rel_{15min}: Drug release in 15 minutes, MDT: Mean dissolution time, DE_{15min}:

Table S5. Composition of solid SNEDDS of OMT prepared using porous carriers

Porous carriers	Amt. of carrier (mg)[$]	True density ($g.cm^{-3}$)	Bulk density ($g.cm^{-3}$)	Tapped density ($g.cm^{-3}$)
Aerosil 200	450	1.67 ± 0.2	0.27 ± 0.12	0.49 ± 0.11
Aeroperl 300	300	1.33 ± 0.3	0.24 ± 0.01	0.31 ± 0.06
Sylysia 550	370	1.29 ± 0.1	0.21 ± 0.07	0.35 ± 0.15
Neusilin US2	250	1.31 ± 0.6	0.20 ± 0.09	0.21 ± 0.05
Fujicalin SG	420	1.44 ± 0.2	0.27 ± 0.04	0.34 ± 0.08

CQA: Critical quality attributes, OMT: Olmesartan medoxomil, SNEDDS: Self-naoemulsifying drug delivery systems

Table S6. Micromeritic properties and drug content estimation of OMT solid SNEDDS

Carriers	Angle of repose (θ)	Carr’s index	Hausner’s ratio
Aerosil 200	37.25 ± 0.87	36.78 ± 0.76	1.58 ± 0.03
Aeroperl 300	29.15 ± 0.85	22.36 ± 1.19	1.28 ± 0.04
Sylysia 550	36.40 ± 1.09	39.52 ± 1.03	1.65 ± 0.04
Neusilin US2	22.65 ± 1.13	17.28 ± 0.58	1.20 ± 0.03
Fujicalin SG	39.37 ± 1.09	21.70 ± 0.70	1.27 ± 0.03

OMT: Olmesartan medoxomil, SNEDDS: Self-naoemulsifying drug delivery systems

Table S7. Effect of accelerated stability studies at 40 ± 2°C/75 ± 5% RH on the formulation parameters

Time (months)	T_{emul} (sec)	Globule size (D_{nm})	Zeta potential (mV)	Drug release (Rel_{15min})
**Optimized liquid SNEDDS**				
0	149.23	71.23	23.45	89.34
1	148.45	72.75	23.56	88.45
2	147.91	73.91	23.49	88.01
3	147.34	74.56	23.87	87.23
6	147.01	74.98	23.91	87.43
**Optimized solid SNEDDS**				
0	150.76	72.13	24.29	85.23
1	151.53	73.39	24.56	84.98
2	152.89	74.17	25.10	84.45
3	153.55	74.89	25.27	83.79
6	154.42	75.22	25.45	83.12

T_{emul}: Emulsification time, Rel_{15min}: Drug release in 15 minutes, SNEDDS: Self-naoemulsifying drug delivery systems
